# The CB1 receptor: linking impulsivity and substance use disorder

**DOI:** 10.3389/fnins.2025.1621242

**Published:** 2025-09-30

**Authors:** Octavio Amancio-Belmont, Lorena Alline Becerril-Melendez, Mónica Méndez-Díaz

**Affiliations:** ^1^Laboratorio de Ontogenia y Adicciones, Departamento de Fisiología, Facultad de Medicina, Universidad Nacional Autónoma de Mexico, México City, Mexico; ^2^División de Neurociencias, Instituto de Fisiología Celular, Universidad Nacional Autónoma de Mexico, México City, Mexico

**Keywords:** impulsivity, CB1 receptor, substance use disorder, adolescent, hippocampus, cerebellum

## Abstract

The cannabinoid receptor type 1 (CB1R) is the most widely expressed G protein-coupled receptor in the brain, with high concentrations in the basal ganglia, hippocampus, and cerebellum. Predominantly localized in presynaptic terminals, CB1R modulates synaptic transmission through retrograde endocannabinoid signaling. Its expression follows an ontogenetic trajectory, with region- and age-specific patterns that are particularly dynamic during adolescence. This developmental window is characterized by marked neuroplasticity and heightened impulsivity, a trait closely associated with increased vulnerability to substance use disorders (SUDs). While the prefrontal cortex has traditionally been viewed as the primary locus of self-control, growing evidence supports a broader regulatory network involving CB1R-rich subcortical structures. In particular, the hippocampus and cerebellum contribute to the modulation of impulsive behavior through their connectivity with prefrontal and limbic circuits. CB1R signaling in these regions influences decision-making, reward sensitivity, and response inhibition—all processes critical to the emergence of impulsive traits and drug-seeking behavior. This review integrates preclinical and clinical evidence to propose a distributed CB1R-regulated neurocircuit that underlies impulsivity and mediates risk for SUDs. We highlight adolescence as a critical period during which CB1R ontogeny may transiently unbalance inhibitory control systems, creating a neurobiological substrate for risky behaviors. Furthermore, we identify key knowledge gaps, including the underexplored ontogeny of CB1R in the cerebellum and its functional implications in addiction vulnerability. Understanding the dynamic role of CB1R across development and brain regions offers a more comprehensive model of impulsivity and its pathological escalation into substance use. This perspective may inform translational strategies targeting the endocannabinoid system for early prevention.

## Introduction

The endocannabinoid system (ECS) regulates emotional, motivational, and cognitive processes associated with impulsivity and drug addiction. Within this system, the cannabinoid type-1 receptor (CB1R) plays an important role due to its widespread expression in brain regions involved in these functions, including the prefrontal cortex (PFC), hippocampus, basal ganglia, amygdala, and cerebellum (Herkenham et al., 1990; Mackie, 2005; Tsou et al., 1998). This distribution suggests that CB1R coordinates cortical and subcortical mechanisms involved in impulse control and drug addiction.

Accumulating evidence indicates that CB1R contributes to impulsive behavior and may increase susceptibility to substance use disorders (SUDs) (Cinnamon Bidwell et al., 2013; Romero-Torres et al., 2023; Schneider et al., 2015). Moreover, age-dependent differences in CB1R expression and function have been observed (Amancio-Belmont et al., 2017; Meyer et al., 2018), potentially contributing to increased impulsivity during key developmental periods.

Impulsivity is a multidimensional trait encompassing poor inhibitory control, premature responding, and maladaptive decision-making. These dimensions have been consistently associated with the initiation of drug use, a higher vulnerability to developing SUDs, early dropout from treatment, and more frequent relapses in individuals (Ersche et al., 2010; Stevens et al., 2014; Verdejo-García et al., 2008). Adolescence represents a critical neurodevelopmental period characterized by elevated impulsive behavior, which may increase the risk of initiating substance use and developing SUDs (Jordan and Andersen, 2017).

This review aims to synthesize recent findings on the role of CB1R in impulsivity, with a particular emphasis on its contribution to SUDs vulnerability. We examine both preclinical and clinical evidence linking CB1R function to impulsivity-related behaviors, highlighting age-dependent differences and therapeutic potential.

## CB1 receptor: expression and function

Since the 1990s, evidence has revealed the existence of an endogenous cannabinoid system (ECS) in the brain and the periphery. This ECS is composed of endogenous cannabinoids, of which the most studied are anandamide and 2 arachidonoyl glycerol (Devane et al., 1992; Hanus et al., 2001; Mechoulam et al., 1995), enzymes for the synthesis [N-acyl-phosphatidylethanolamine (NAPE)], diacylglycerol (DAG) and degradation [fatty acid amide hydrolase (FAAH), monoacylglycerol lipase (MAGL)], and cannabinoids receptors CB1R and CB2R, which mediate their actions (Devane et al., 1988; Matsuda et al., 1990; Munro et al., 1993). Agonist stimulation of both receptors activates several transduction pathways via the Gi/o family of G proteins-coupled signaling cascades, leading to inhibition of neurotransmitter release (Howlett et al., 2002).

CB1R is highly concentrated at presynaptic terminals, where it mediates retrograde signaling of endocannabinoids (Katona et al., 1999; Tsou et al., 1998). In addition to neurons, CB1R is expressed, although to a much lesser extent, in astrocytes, oligodendrocytes, and microglia (Stella, 2009). The basal ganglia, hippocampus, and cerebellum are the structures exhibiting the highest levels of CB1R expression (Herkenham et al., 1990; Matsuda et al., 1993). CB1R is predominantly localized at the presynaptic terminals of cholecystokinin-expressing (CCK) GABAergic interneurons and certain glutamatergic neurons (Katona et al., 1999). This distribution enables CB1R to modulate both inhibitory and excitatory synaptic transmission. Additionally, CB1R is present in glutamatergic neurons, albeit at lower levels, contributing to the fine-tuning of excitatory neurotransmission (Hill et al., 2007; Katona et al., 1999; Marsicano and Lutz, 1999). This dual localization highlights the crucial role of CB1R in maintaining the balance between excitation and inhibition in neural circuits.

CB1R activity is involved in several physiological functions, including neuronal development (Fride, 2008), coordination and control of movement (Giuffrida and Piomelli, 2000), stress response (Beins et al., 2021), food intake (Kirkham et al., 2002; Méndez-Díaz et al., 2015), regulation of sleep (Méndez-Díaz et al., 2013; Prospéro-García et al., 2016), body temperature (Smirnov and Kiyatkin, 2008), pain (Yang et al., 2016), immune function (Pandey et al., 2009), reward (Méndez-Díaz et al., 2019; Parsons and Hurd, 2015) and higher cognitive functions, especially those related to learning and memory (Marsicano and Lafenêtre, 2009). Emerging evidence suggests a role for CB1R in behavioral regulation, including inhibitory control and decision-making.

In mesolimbic circuits, CB1R modulates dopaminergic activity, particularly in the ventral tegmental area (VTA) and nucleus accumbens (NAcc). This modulation enhances the reinforcing effects of both natural rewards (Amancio-Belmont et al., 2017; Méndez-Díaz et al., 2012) and drug of abuse (Amancio-Belmont et al., 2019; Castañé et al., 2002; Manzanedo et al., 2004), contributing to motivation and goal-directed behavior, but also to pathological reinforcement processes underlying drug-seeking and addiction such as drug-seeking and drug addiction (Peters et al., 2020). CB1R also influences stress-coping mechanisms by modulating hypothalamic–pituitary–adrenal (HPA) axis activity. Under stress, endocannabinoid signaling increases, promoting neuroendocrine and behavioral adaptation by dampening glutamatergic excitability and reducing anxiety-like behaviors (Morena et al., 2016).

## Impulsivity

**Figure 1:**
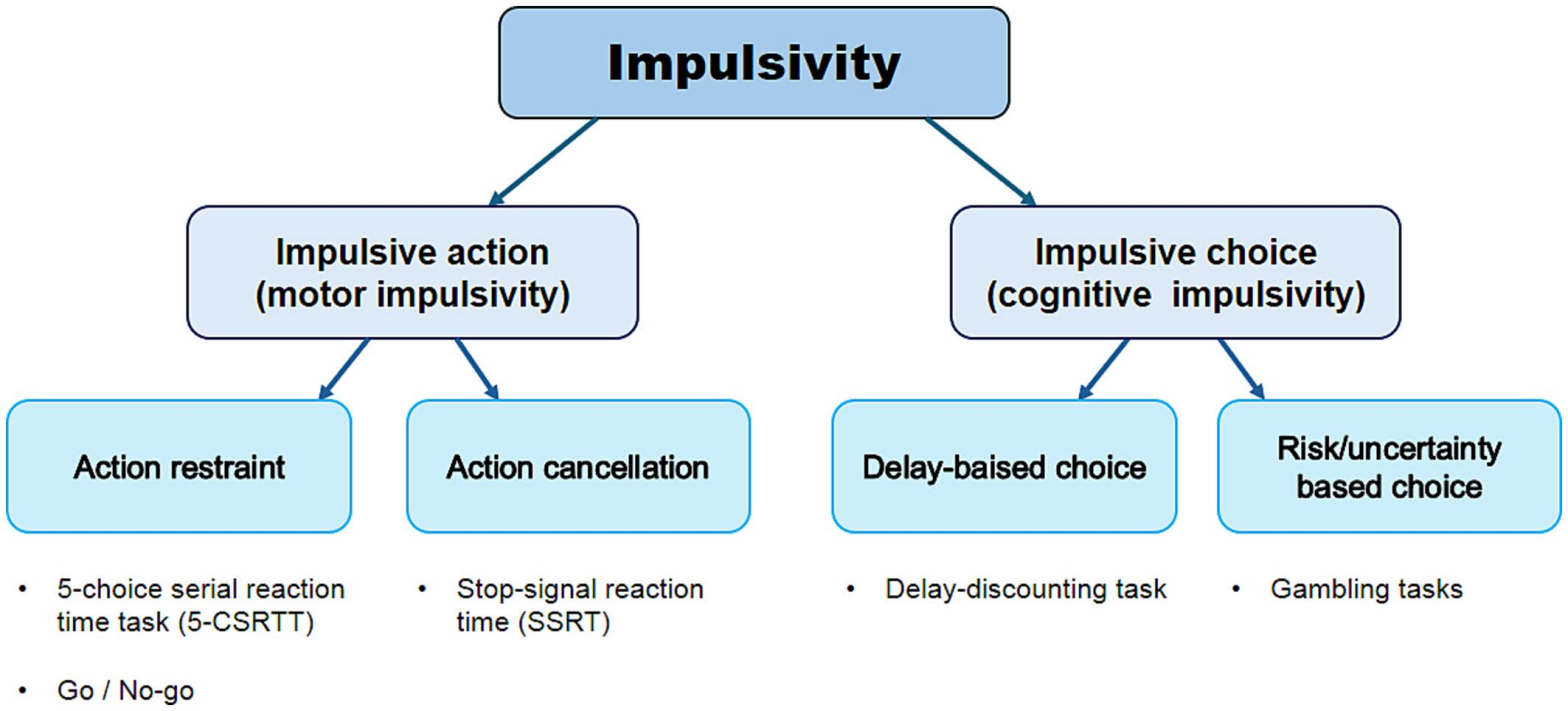
Overview of different modalities of impulsive behavior and neuropsychological tasks used to measure them. Impulsivity is typically conceptualized in two distinct forms: impulsive action, which refers to a failure of behavioral inhibition despite potential negative consequences, and impulsive choice, which involves a deficit in self-control or an inability to delay gratification.

Impulsivity is defined as a predisposition toward rapid, unplanned reactions in response to internal or external stimuli, often yielding negative consequences (De Wit, 2009; Gell et al., 2024; Peyton et al., 2019). The ability to make rapid decisions without hesitation can be advantageous in certain situations, but impulsive behavior is generally maladaptive in everyday life (Peyton et al., 2019). Impulsivity is a broad, multifaceted construct typically described in two ways: (1) impulsive action, defined as a lack of behavioral inhibition regardless of potential negative consequences, and (2) impulsive choice, defined as failure of self-control or inability to delay gratification (Figure 1) (Grant and Chamberlain, 2014; Weafer et al., 2014). Impulsivity plays a crucial role in human behavior and is closely linked to cognitive control and decision-making processes (Dalley et al., 2011).

The ability to regulate emotions and behaviors in the face of temptation or impulses is known as self-control, involves delaying gratification, resisting immediate urges, and focusing on long-term goals (Duckworth and Steinberg, 2015) and depends critically of the PFC (Dalley et al., 2002; Euston et al., 2012; Friedman and Robbins, 2022; Kim and Lee, 2011). Therefore, the culmination of PFC development during adulthood contributes to rational decision-making, which is largely based on self-control. In this context, adults are expected to display fewer impulsive actions in daily life (Harden and Tucker-Drob, 2011). When adults consistently make impulsive choices or risky decisions, these may be maladaptive and even symptomatic of psychiatric disorders, such as SUDs (De Wit, 2009; Méndez Díaz et al., 2021; Raji et al., 2025).

Adolescents, however, often exhibit impulsive behavior driven by a desire for sensation, novelty, and adventure, leading to increased risk-taking and heightened reward sensitivity (Doremus-Fitzwater and Spear, 2016; Harden and Tucker-Drob, 2011). In a longitudinal study with a two-year follow-up, sensation seeking significantly mediated the effect of resting-state hyperconnectivity (particularly in prefrontal, medial frontoparietal, and occipitotemporal networks) on subsequent alcohol use. These findings highlight the role of connectivity within and between inhibitory control and reward-processing networks in contributing to facets of impulsivity and risk for early substance use initiation among adolescents (Antón-Toro et al., 2022).

Adolescent alcohol misuse (AAM) has been associated with disruptions in brain development. A longitudinal machine learning analysis of the IMAGEN dataset (n = 1,182) identified neuroanatomical features in adolescence that predicted AAM. Specifically, reduced white matter integrity in the corpus callosum, internal capsule, and brainstem, as well as alterations in gray matter in the occipital, frontal, and temporal lobes, as well as the cingulate cortex, were significant predictors of later alcohol misuse (Rane et al., 2022). These findings support the notion that adolescence represents a critical window of vulnerability, during which structural immaturity os circuits for impulse control, reward, and cognitive regulation confers risk for SUDs.

Impulsivity has been consistently described in adolescent rodents. Using several experimental paradigms, Andrzejewski et al. (2011) found that adolescent rats display impaired learning, reduced behavioral inhibition, and lower self-control compared to adult rats. Additionally, both female and male adolescent rats exhibit more impulsive actions than their adult counterparts (Burton and Fletcher, 2012). Adolescent mice also show increased impulsivity in tasks such as the Five-Choice Serial Reaction Time Task (5-CSRTT) (Ciampoli et al., 2017) and the Delayed Delivery Task (Adriani and Laviola, 2003). Moreover, adolescent rats exhibit rapid consumption patterns and an increased eagerness to seek out palatable food (Amancio-Belmont et al., 2017; Friemel et al., 2010). In this context, heightened reward sensitivity may also generalize to drug intake, as natural reinforcers and drugs of abuse engage overlapping neurobiological pathways. For example, low doses of alcohol, nicotine, cocaine, and amphetamine produce conditioned place preference in adolescents, but not adults, suggesting that adolescents may be more sensitive to the rewarding effects of drugs of abuse (Brenhouse et al., 2008; Philpot et al., 2003; Vastola et al., 2002; Zakharova et al., 2009).

Although impulsivity is often conceptualized as a failure of self-regulation, it may be more accurately conceptualized as the result of imbalances or inefficiencies within the neural circuits that support self-control. While the PFC remains central, growing evidence highlights the contribution of subcortical partners (Méndez Díaz et al., 2021). The PFC exerts top-down executive control through extensive bidirectional connections with structures such as the basal ganglia, which are involved in action selection and motor inhibition, mainly via the dorsal striatum (Aron et al., 2003; Terra et al., 2020). Thalamocortical pathways support these cortico-striatal loops, which enhance response suppression and attentional control (Alexander and Crutcher, 1990; Aron and Poldrack, 2006). The amygdala contributes emotional salience, influencing behavior in affectively charged contexts (Guex et al., 2020), while the VTA modulates reward-driven behaviors through dopaminergic projections to the PFC and NAcc (Flores-Dourojeanni et al., 2021; Han et al., 2017). Together, these regions form an integrated cortico-subcortical network essential for delaying gratification, suppressing impulsive tendencies, and promoting adaptive, goal-directed behavior.

## CB1 receptor and impulsivity

Experimental evidence demonstrates that CB1R regulates impulsivity. In humans, the systemic administration of Δ^9^-Tetrahydrocannabinol (THC) increases impulsive responding on the Stop task (McDonald et al., 2003). In rodents, systemic administration of SR141716A, a CB1R antagonist, enhanced inhibitory control by decreasing the number of premature responses in the 5-CSRTT in rats (Pattij et al., 2007). Chronic exposure to WIN 55,212–2, a CB1R agonist, during adolescence increased impulsive choice in delay-discounting tasks: adolescent-onset administration produced a marked preference for smaller, immediate rewards, whereas adult-onset exposure had a much weaker effect, underscoring adolescence as a critical window of vulnerability to cannabinoid-induced deficits in self-control (Johnson et al., 2019).

Research further supports the link between CB1R signaling and impulse control, particularly through evidence from pathological models that demonstrate how CB1R upregulation affects behavioral inhibition. In a study involving a Tat transgenic mouse model of HIV-associated neurocognitive disorders (HAND), female mice expressing the viral Tat protein exhibited deficits in inhibitory control during a Go/No-Go task. These impairments were accompanied by increased CB1R expression in the infralimbic cortex, a subregion of the PFC that plays a crucial role in behavioral regulation. Importantly, higher levels of CB1R expression were associated with poorer task performance, suggesting that CB1R overexpression may impair top-down inhibitory processes, even outside the context of typical neurodevelopment (Jacobs et al., 2019).

The developmental trajectory of CB1R expression shows marked age-dependent variation. Western blot and immunohistochemistry studies reveal that adolescent rodents exhibit lower CB1R expression in the medial prefrontal cortex (mPFC), hippocampus (dorsal and ventral), and NAcc compared to adults and aged animals (Amancio-Belmont et al., 2017) while showing relatively higher levels in specific dorsal hippocampal subfields such as CA3 and dentate gyrus (Romero-Torres et al., 2023).

These findings are particularly relevant when considered in the context of adolescence, a developmental window characterized by naturally elevated impulsivity and an immature prefrontal regulatory system. During this period, CB1R expression undergoes dynamic changes, and even subtle disruptions in its signaling, whether due to genetic, pharmacological, or environmental factors, can have lasting effects on impulse control. Given the heightened plasticity and ongoing maturation of front-cortical and subcortical networks during adolescence, increased CB1R expression in key regions may exacerbate existing vulnerabilities. This supports the notion that adolescent brains are not only more reactive to endocannabinoid modulation but also more susceptible to its dysregulation (Cass et al., 2014), potentially contributing to long-term behavioral disinhibition and an increased risk of substance use.

## Impulsivity as an adaptive feature in adolescence

Although impulsivity is often framed as a risk factor for maladaptive behaviors (particularly substance use), it also serves important adaptive functions during adolescent development. From an evolutionary perspective, increased impulsivity and risk-taking in adolescence may have conferred advantages by promoting novelty-seeking, exploration of new environments, and the pursuit of independence from parental figures (Ellis et al., 2012; Spear, 2000). These behaviors are essential for acquiring new skills, forming social connections, and developing survival strategies necessary for adulthood.

Within this framework, the transient configuration of CB1R expression and endocannabinoid signaling during adolescence should not be pathologized but instead understood as part of a broader neurodevelopmental strategy. Endocannabinoid signaling plays a key role in modulating synaptic plasticity, emotional regulation, and the maturation of prefrontal-limbic circuitry during this critical developmental period (Meyer et al., 2018; Tseng and Molla, 2025).

The challenge arises when these adaptive behaviors occur in environments saturated with immediate rewards and risks, such as easy access to psychoactive substances, lack of adult guidance, or emotionally unstable contexts, where impulsive tendencies can quickly escalate into maladaptive and harmful patterns (Casey et al., 2011; Chambers et al., 2003; Steinberg, 2008).

Therefore, rather than attempting to suppress impulsivity entirely, a more constructive approach involves creating supportive environments, in families, schools, and communities, that help adolescents channel their exploratory drive into safe and enriching activities (Carvalho et al., 2023; Joo and Lee, 2020; Khurana and Romer, 2020). Recognizing impulsivity as a developmentally appropriate trait and focusing on strengthening self-regulatory scaffolding through education, emotional support, and positive reinforcement may reduce the risk of impulsivity contributing to long-term negative outcomes (Carvalho et al., 2023; Zelazo and Carlson, 2012).

## CB1 receptor and substance use disorder

The cannabinoid type 1 receptor (CB1R) plays a central role in the neurobiology of SUDs, given its modulatory influence on reward processing, reinforcement learning, and motivation (Parsons and Hurd, 2015; Serrano and Parsons, 2011). Preclinical studies have shown that activation of CB1R increases alcohol intake in a two-bottle choice paradigm (Colombo et al., 2002), increases morphine-induced CPP (Manzanedo et al., 2004), and reinstates nicotine and cocaine-seeking behavior (De Vries et al., 2001; Gamaleddin et al., 2012).

In contrast, pharmacological blockade of CB1R with antagonists such as rimonabant or AM251 has been shown to reduce alcohol, nicotine, morphine, methamphetamine, and heroin self-administration (Caille et al., 2007; Caillé and Parsons, 2003; Cohen et al., 2002; Economidou et al., 2006; Vinklerová et al., 2002). Also, CB1R blockade significantly attenuates motivation for cocaine self-administration under a progressive ratio schedule of reinforcement (Soria et al., 2005; Xi et al., 2008) and reduces alcohol intake in a two-bottle choice paradigm (Colombo et al., 1998). Moreover, CB1R blockade prevents nicotine and morphine-induced CPP (Le Foll and Goldberg, 2004; Manzanedo et al., 2004) and reduces cocaine-induced CPP (Delis et al., 2017). In addition, CB1R blockade attenuates cocaine- and cue-induced reinstatement of cocaine-seeking behavior, but not stress-induced reinstatement (De Vries et al., 2001). Also, reduces nicotine cue-induced reinstatement of nicotine-seeking behavior (Gamaleddin et al., 2012). Additionally, CB1R KO mice reduce alcohol intake in a two-bottle choice paradigm, prevent alcohol-induced CPP (Houchi et al., 2005; Thanos et al., 2005), and nicotine-induced CPP (Castañé et al., 2002). Also, CB1R KO mice have lower motivation for cocaine self-administration under a progressive ratio schedule of reinforcement (Soria et al., 2005). Furthermore, using non-maternal care deprivation and maternal care deprivation rats, higher alcohol consumption in a two-bottle choice paradigm was correlated with high CB1R expression in the NAcc and low CB1R expression in the mPFC (Amancio-Belmont et al., 2019). These findings support the view that CB1R contributes not only to the initiation of substance use but also to the maintenance and relapse stages of addiction.

Importantly, CB1R expression exhibits developmental plasticity, with adolescence representing a period of enhanced susceptibility to drug-induced neuroadaptations. Exposure to cannabinoids during adolescence can lead to persistent changes in CB1R expression, CB1R signaling, and synaptic plasticity (Burston et al., 2010; Rubino et al., 2009; Weed et al., 2016), particularly in regions involved in impulse control and reward processing (Miller et al., 2019). For instance, adolescent cannabinoid exposure has been associated with increased impulsive decision-making (Cajiao-Manrique et al., 2023; Dougherty et al., 2013), altered dopaminergic function (Solinas and Melis, 2024), and greater vulnerability to subsequent drug use, even when the initial exposure is limited to this developmental window (Orihuel et al., 2021).

Furthermore, clinical and postmortem studies in individuals with SUDs have revealed dysregulation of the ECS, including altered CB1R expression in prefrontal and limbic regions (Hirvonen et al., 2012; Endorzain et al., 2015). These alterations may reflect both pre-existing vulnerabilities and neuroadaptive responses to chronic substance use. Emerging evidence suggests that individual differences in CB1R function influenced by genetic variations, early-life experiences, or developmental stages may increase the risk of SUDs by impacting traits such as impulsivity, reward sensitivity, and stress responsiveness (Haughey et al., 2008; Schacht et al., 2012; Amancio-Belmont et al., 2020; Romero-Torres et al., 2023).

Altogether, this body of evidence supports a model in which CB1R acts as a molecular gateway linking impulsive behavior and substance use vulnerability. Given its positioning at the interface of executive control, affective processing, and motivational systems, CB1R may play a critical role in the neurobiological underpinnings of addiction (Parsons and Hurd, 2015; Zapata and Lupica, 2021).

## The hippocampus and cerebellum in impulsivity and SUDs

While most research has focused on prefrontal-limbic circuits, emerging evidence indicates that additional CB1R-rich regions, such as the hippocampus and cerebellum, play a complementary role in linking impulsivity with substance use vulnerability. These structures, through their connections with executive and motivational networks, provide an expanded framework for understanding the neurobiology of addiction.

The hippocampus is traditionally known for its roles in learning, memory, and spatial navigation. However, it also plays a key role in behavioral flexibility and the integration of contextual and emotional cues that affect decision-making. When hippocampal function is disrupted (Abela and Chudasama, 2014), it can lead to difficulties in evaluating long-term outcomes, which may result in more impulsive choices. Notably, research has found that adolescent rats have higher levels of CB1R expression in the dorsal and ventral hippocampus compared to adults (Amancio-Belmont et al., 2017). This difference may contribute to changes in reward processing and increased impulsivity during adolescence.

Recent research has further elucidated the role of the hippocampus in impulsivity and substance use behaviors. In a study by Romero-Torres et al. (2023), adolescent rats exhibited higher impulsivity in a delay discounting task compared to adults, alongside increased alcohol consumption and alcohol-CPP. Notably, these behavioral tendencies correlated with elevated CB1R expression in the CA3 and dentate gyrus regions of the dorsal hippocampus, suggesting a significant involvement of hippocampal CB1R in modulating impulsive actions and alcohol-seeking behaviors during adolescence.

The cerebellum, traditionally viewed as a motor coordination center, has recently been implicated in higher-order functions, including cognitive and affective regulation. Cerebellar circuits interact with prefrontal and limbic regions via cerebello-thalamo-cortical and cerebello-striatal pathways(Palesi et al., 2017; Watson et al., 2014), supporting functions such as temporal prediction, response inhibition, and reward expectancy (Kostadinov and Häusser, 2022; Mannarelli et al., 2020). CB1R is expressed in Purkinje cells and cerebellar interneurons (Ashton et al., 2004), where it modulates synaptic transmission and timing precision. Preclinical studies indicate that cerebellar CB1R activity may influence impulsive behavior by regulating the temporal dynamics of decision-making and motor output. For example, cerebellar disruption has been linked to premature responses and poor delay discounting performance, hallmarks of impulsive behavior (Miquel et al., 2019; Murphy et al., 2017; Ortiz et al., 2015).

Despite the increasing interest in the role of the cerebellum in behavioral regulation, little is known about the ontogeny of CB1R expression within cerebellar circuits. This knowledge gap is significant, especially considering the cerebellum’s growing importance in impulsivity and vulnerability to substance use. Understanding how the developmental trajectories of cerebellar CB1R contribute to the maturation of self-control and the neurobiological mechanisms underlying SUDs could provide valuable insights.

Incorporating these regions into more comprehensive models of impulsivity and SUDs could help clarify behavioral phenomena that prefrontal mechanisms alone do not fully explain, such as impulsive actions in neutral contexts or rapid shifts between goal-directed and habitual behavior. We proposed integrating the cerebellum into broader circuits of self-control, emphasizing its anatomical connectivity with prefrontal and subcortical regions.

## The role of CB1R in adolescence: developmental considerations

Adolescence is a unique period of neurodevelopment characterized by significant structural and functional changes in the brain. These changes include synaptic pruning, myelination, and the maturation of cortico-subcortical circuits (Arain et al., 2013; Juraska and Drzewiecki, 2020). Alongside these changes, there are fluctuations in endocannabinoid signaling, particularly involving the expression and functionality of the CB1R. The expression of CB1R follows an ontogenetically regulated pattern, with region-specific changes that peak at different stages (de Fonseca et al., 1993; Mato et al., 2003). Research in rodents has indicated that CB1R expression is elevated in various brain regions during early adolescence but gradually decreases into adulthood, particularly in the PFC and hippocampus (Heng et al., 2011; Meyer et al., 2018).

This transient elevation of CB1R, during adolescence may reflect the system’s role in shaping synaptic connectivity and regulating excitatory-inhibitory balance during critical periods of plasticity, making this stage particularly vulnerable to external insults. CB1R density is especially high in prefrontal, hippocampal, and cerebellar circuits, which are still undergoing synaptic refinement. Exogenous activation of CB1R during this period can disrupt excitatory-inhibitory balance and interfere with the maturation of executive functions and impulse control, ultimately leading to persistent cognitive and behavioral alterations (Schneider, 2008). Cannabis, one of the most commonly used drugs during adolescence, has been shown to impair cognitive processes such as short-term memory function or attention (Sullivan, 2000). Neuroimaging studies revealed that adolescent cannabis users exhibit a lower percentage of gray matter and a higher percentage of white matter relative to whole brain volume (Pope et al., 2003). Cannabis use has also been associated with increased vulnerability to other drugs of abuse, such as opioids (Ellgren et al., 2007). In line with these findings, preclinical studies demonstrate that adolescent cannabinoid exposure produces enduring impairment in working memory, behavioral flexibility, and response inhibition in adulthood (Molla and Tseng, 2020; Renard et al., 2018).

Moreover, CB1R activity during adolescence appears to shape the development of motivational systems. Enhanced CB1R signaling in limbic and mesolimbic areas may amplify reward sensitivity and novelty-seeking behavior, traits that are adaptive for exploration but may also increase susceptibility to risk-taking and substance use, especially in adverse environments (Renard et al., 2016; Schneider et al., 2015).

Taken together, these findings emphasize that the adolescent ECS is not dysfunctional, but developmentally distinct. The typical ontogeny of CB1R expression and function may itself represent a neurobiological substrate of vulnerability to impulsive behavior and substance use. Understanding this temporally dynamic profile is essential for identifying sensitive periods in which interventions may be most effective for promoting self-regulation and preventing addiction.

These findings support a more distributed view of impulse control, in which hippocampal and cerebellar networks, through their interactions with CB1R, shape behavioral outcomes relevant to substance use. Yet, this distributed model does not diminish the central role of the PFC. While the PFC has traditionally been regarded as the primary node for executive control, its relevance becomes particularly evident during adolescence, when this region undergoes protracted maturation. Together with hippocampal and cerebellar contributions, these interactions highlight a broader network in which CB1R influences developmental trajectories of impulse control and vulnerability to SUDs.

The hippocampus, with its dense CB1R innervation, participates not only in memory formation but also in the contextual evaluation of actions and outcomes. CB1R signaling in the hippocampus modulates behavioral flexibility and decision-making under uncertainty, processes that are core to impulsive control (Abush and Akirav, 2010).

The cerebellum is now recognized as a contributor to cognitive and affective regulation. CB1R signaling is critical for long-term depression (LTD) at parallel fiber–Purkinje cell synapses, a mechanism essential for motor learning and sensorimotor adaptation (Safo and Regehr, 2005). Through the synchronized activity of Purkinje cells and cerebellar nuclei neurons, cerebellar circuits enable accurate timing and anticipatory control of both motor and cognitive outputs (Person and Raman, 2012). Its afferent and efferent connections with midbrain and limbic regions provide the neuroanatomical basis for involvement in emotional regulation (Schutter and Van Honk, 2005), as exemplified in the cerebellar cognitive affective syndrome (CCAS), characterized by deficits in executive function and affect modulation (Schmahmann, 2019). Recent anatomical and functional evidence demonstrates that the cerebellum may directly influence reward-related midbrain circuits: in mice, optogenetic activation of excitatory projections from the deep cerebellar nuclei to the VTA promotes reward-seeking behavior, underscoring a cerebello–VTA pathway that contributes to reinforcement (Carta et al., 2019). This connectivity suggests a potential route by which cerebellar CB1R signaling could modulate mesolimbic dopamine activity, thereby indirectly influencing impulsivity and vulnerability to substance use disorders. Overall, these findings support the view that the cerebellum may fine-tune impulsive responses through modulation of prefrontal and limbic outputs.

However, the ontogenetic CB1R expression in the cerebellum and its relationship to behavioral outcomes remain largely unexplored. This knowledge gap is of particular interest to our laboratory and represents a focus of our ongoing research.

## An integrative model of CB1R and impulsivity

Converging clinical and preclinical evidence supports an integrative model wherein CB1R modulates impulsivity by affecting both cortical control systems, such as PFC, and subcortical evaluative networks, including the hippocampus, cerebellum. This expanded neurobiological framework moves beyond the focus on the PFC and aligns with modern models of distributed executive function. Additionally, it highlights the significance of developmental periods, such as adolescence, during which alterations in CB1R signaling may lead to persistent changes in self-regulation and behavioral trajectories associated with SUDs.

Building on this well-established cortico-subcortical framework, we propose that other brain structures, specifically the hippocampus and the cerebellum, may play a more integral role in the regulation of self-control than initially recognized. The hippocampus, through its connections with the VTA, amygdala, and PFC, provides contextual and mnemonic input that is critical for evaluating action-outcome contingencies and delaying responses. Similarly, the cerebellum, which projects to the PFC and basal ganglia via thalamic relays, has been increasingly implicated in the modulation of cognitive timing, error prediction, and inhibitory motor control. The high density of CB1R in these regions further supports their potential involvement in the endocannabinoid modulation of impulsivity.

Taken together, we suggest that the hippocampus and cerebellum, which have traditionally been overlooked in models of impulse regulation, may be part of a broader, distributed circuit that contributes to both cognitive and motor aspects of self-control. Recognizing these extended interactions may enhance our understanding of the neurobiological mechanisms underlying impulsivity and expand the scope of future interventions.

## Therapeutic perspectives and challenges in CB1R modulation

While CB1R remains a promising target for modulating impulsivity and, by extension, vulnerability to SUDs, direct pharmacological manipulation presents significant challenges. CB1R antagonists or inverse agonists have shown potential in reducing drug-seeking behavior and improving cognitive control in preclinical models; however, their clinical translation has been hindered by psychiatric side effects, including anxiety and depression, as observed in trials with rimonabant (Christensen et al., 2007; Vinod and Hungund, 2006).

Efforts to develop CB1R antagonists without the psychiatric side effects observed with inverse agonists such as rimonabant are ongoing. The neutral antagonist AM6527 has shown efficacy in reducing drug-seeking behavior in preclinical models while avoiding aversive or depressive-like effects (Soler-Cedeño et al., 2024), but remains under investigation and has not yet progressed to clinical trials.

Given these limitations, a more viable avenue may involve indirect modulation of CB1R signaling to enhance behavioral self-regulation. It has been demonstrated that developing self-control during childhood and adolescence is associated with improved academic achievement, healthier social relationships, and reduced risk-taking behaviors across the lifespan, like drug abuse (Diamond and Lee, 2011; Duckworth et al., 2019).

## Conclusion

CB1R emerges as a key neuromodulator element linking impulsivity and vulnerability to SUDs, particularly during adolescence, a developmental period marked by dynamic changes in endocannabinoid signaling. While traditionally centered on the PFC, impulse control involves a broader network that includes the hippocampus and cerebellum, both of which are enriched in CB1R and undergo significant maturation during adolescence. Understanding how CB1R contributes to the regulation of self-control across cortical and subcortical regions offers a more integrative framework for identifying neurobiological risk factors and potential intervention targets for impulsivity-driven behaviors and addiction.
